# Group IV Getah Virus in *Culex* Mosquitoes, Malaysia

**DOI:** 10.3201/eid2802.204887

**Published:** 2022-02

**Authors:** Sing-Sin Sam, Noor-Adila Mohamed-Romai-Noor, Boon-Teong Teoh, Zur-Raiha Hamim, Hoi-Ying Ng, Juraina Abd-Jamil, Chee-Sieng Khor, Sharifah-Syed Hassan, Hamdan Ahmad, Sazaly AbuBakar

**Affiliations:** Universiti Malaya, Kuala Lumpur, Malaysia (S.-S. Sam, N.-A. Mohamed-Romai-Noor, B.-T. Teoh, Z.-R. Hamim, H.-Y. Ng, J. Abd-Jamil, C.-S. Khor, S. AbuBakar);; Monash University Malaysia, Selangor, Malaysia (S.-S. Hassan);; Universiti Sains Malaysia, Pulau Pinang, Malaysia (H. Ahmad)

**Keywords:** Getah virus, mosquitoes, viruses, infectious diseases, tropical, emerging, vector-borne infections, arbovirus, alphavirus, evolution, Malaysia

## Abstract

A new Getah virus (GETV) strain, B254, was isolated from *Culex fuscocephalus* mosquitoes captured at Mount Ophir, Malaysia, in 2012. Phylogenetic analyses revealed that GETV B254 is distinct from the old Malaysia GETV MM2021 strain but closely related to group IV GETV from Russia (LEIV16275Mag), China (YN12031), and Thailand (GETV/SW/Thailand/2017).

Getah virus (GETV) is an emerging mosquitoborne alphavirus of the family *Togaviridae*. The virus was first reported in 1955 from *Culex gelidus* mosquitoes collected near Kuala Lumpur, Malaysia (*1*). Serologic evidence of GETV infection was found in various large domestic mammals (*2*) and humans (*3*). GETV infections in these populations, however, were mostly inapparent. In other regions, reproductive failures in pregnant sows, death in piglets, and hind limb edema in racehorses, as well as neurologic symptoms and death in blue foxes, have been reported (*4*–*7*).

We conducted mosquito surveillance in the forests of 4 different states in Peninsular Malaysia, Perak, Pahang, Selangor, and Johor, during 2011–2014. We captured a total of 4,160 mosquitoes from the study sites by BG-Sentinel CO_2_ trap (Biogents AG, https://eu.biogents.com) with the addition of UV light and sorted them into 208 pools according to their species, determined by morphologic keys, and collection sites. We homogenized the pooled mosquitoes, inoculated them into C6/36 mosquito cells, and blind passaged for 5 passages. We obtained cytopathic effects from 1 of the pools on day 4 postinfection during the last passage. The pool comprised *Cx. fuscocephalus* mosquitoes collected from the forested area at Mount Ophir (Malay: Gunung Ledang) in Johor in 2012. We screened the culture supernatants by reverse transcription PCR (RT-PCR) using the in-house developed alphavirus generic primers targeting the nsP4 gene and flavivirus primers targeting the nonstructural 5–3′ untranslated region junction. A similar sample pool that showed cytopathic effects was found positive for the presence of alphaviruses, as indicated by a 683 bp amplicon. Sanger sequencing of the amplicon revealed high sequence similarity to GETV. We obtained the complete coding sequence of the GETV isolate, designated as B254, using 10 pairs of RT-PCR amplification primers and genome sequencing. We deposited the genome in GenBank (accession no. LR990838).

We constructed a maximum clade credibility tree using the Bayesian Markov chain Monte Carlo analysis based on the complete coding regions of GETV B254 and other GETV isolates available in GenBank (Figure). The GETV phylogeny revealed 4 major groups of viruses: group I (GI), GII, GIII, and GIV (*8*). GI and GII consisted of early identified GETV isolates; GI contained the Malaysia MM2021 isolate (1955), and GII, Japan Sagiyama (1956). The GIII and GIV comprised the most recent circulating virus strains. The newly isolated Malaysia GETV B254 is phylogenetically distinct from the ancestral Malaysia MM2021. It clustered within the GIV clade, which included the strains recovered in Russia (LEIV16275Mag, 2007), China (YN12031, 2012) and Thailand (GETV/SW/Thailand/2017). The B254 strain showed the highest nucleotide sequence homology with YN12031 (98.8%), isolated from the *Armigeres subalbatus* mosquito, and GETV/SW/Thailand/2017 (98.6%), isolated from pig serum.

**Figure:**
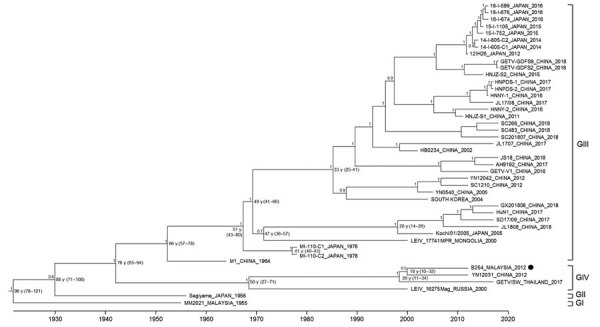
Maximum-clade credibility tree of complete coding sequences of Getah virus (GETV) strain B254 from Malaysia (black dot) and reference strains. Horizontal branches are drawn to a scale of estimated year of divergence. Times to the most recent common ancestor with 95% highest posterior density values (ranges in parentheses) are shown at nodes. Posterior probability values >0.6 of key nodes are shown. G, group.

Currently, only the GIII GETV lineage is associated with epidemic outbreaks. The GIV GETV, however, consisted of only a few virus strains. We calculated the mean evolutionary rate of GIV GETV at 4.10 × 10^−4^ substitutions/site/year and the rate for GIII at 2.98 × 10^−4^ substitutions/site/year. These results suggest that the GIV viruses may be under a different selection pressure, potentially involving a different host. It is also possible that GIV viruses are maintained in a yet-to-be-identified enzootic transmission cycle involving mosquitoes and asymptomatic hosts.

The *Culex* mosquitoes, particularly *Cx. gelidus* and *Cx. tritaeniorhynchus*, have been identified as the most predominant vector host for GETV in Malaysia (*1*,*2*). *Cx. fuscocephalus* mosquitoes, however, are not an uncommon vector; they have been known to carry the virus in China and Sri Lanka (*9*,*10*). *Cx. fuscocephalus* mosquitoes could be an emerging vector for GETV transmission in Malaysia along with the other *Culex* mosquitoes.

The high similarity of genome sequences of GIV GETV strains recovered thousands of kilometers apart raised the possibility that the virus may share a common dispersal route. One possible route linking the virus is the East Asian–Australasian flyway of migratory birds. GIV GETV could have been introduced to the regions through the migratory birds along the flyway, which included Malaysia as one of the stopover sites; the abundantly available *Culex* mosquitoes at the roosting sites could have maintained the transmission.

In summary, we identified a new GETV strain, B254, in Malaysia that is phylogenetically distinct from the old Malaysia MM2021 strain. The virus strain shares high similarities to the widely distributed GIV GETV. Although further surveillance studies are needed for confirmation, this finding suggests that GIV GETV strains could share a common dispersal origin, possibly through the bird transmigratory flyways. 

## References

[1] Simpson DI, Way HJ, Platt GS, Bowen ET, Hill MN, Kamath S, et al. Arbovirus infections in Sarawak, October 1968-February 1970: GETAH virus isolations from mosquitoes. Trans R Soc Trop Med Hyg. 1975;69:35–8. 10.1016/0035-9203(75)90008-5 238314

[2] Marchette NJ, Rudnick A, Garcia R, MacVean DW. Alphaviruses in Peninusular Malaysia: I. Virus isolations and animal serology. Southeast Asian J Trop Med Public Health. 1978;9:317–29. 34888

[3] Marchette NJ, Rudnick A, Garcia R. Alphaviruses in Peninsular Malaysia: II. Serological evidence of human infection. Southeast Asian J Trop Med Public Health. 1980;11:14–23. 7403943

[4] Brown CM, Timoney PJ. Getah virus infection of Indian horses. Trop Anim Health Prod. 1998;30:241–52. 10.1023/A:1005079229232 9760716

[5] Nemoto M, Bannai H, Tsujimura K, Kobayashi M, Kikuchi T, Yamanaka T, et al. Getah virus infection among racehorses, Japan, 2014. Emerg Infect Dis. 2015;21:883–5. 10.3201/eid2105.141975 25898181 PMC4412242

[6] Yang T, Li R, Hu Y, Yang L, Zhao D, Du L, et al. An outbreak of Getah virus infection among pigs in China, 2017. Transbound Emerg Dis. 2018;65:632–7. 10.1111/tbed.12867 29575687

[7] Shi N, Li LX, Lu RG, Yan XJ, Liu H. Highly pathogenic swine Getah virus in blue foxes, eastern China, 2017. Emerg Infect Dis. 2019;25:1252–4. 10.3201/eid2506.181983 31107236 PMC6537705

[8] Li YY, Liu H, Fu SH, Li XL, Guo XF, Li MH, et al. From discovery to spread: The evolution and phylogeny of Getah virus. Infect Genet Evol. 2017;55:48–55. 10.1016/j.meegid.2017.08.016 28827175

[9] Peiris JS, Amerasinghe PH, Amerasinghe FP, Calisher CH, Perera LP, Arunagiri CK, et al. Viruses isolated from mosquitoes collected in Sri Lanka. Am J Trop Med Hyg. 1994;51:154–61. 10.4269/ajtmh.1994.51.154 7915499

[10] Zhang HL, Zhang YZ, Yang WH, Feng Y, Nasci RS, Yang J, et al. Mosquitoes of Western Yunnan Province, China: seasonal abundance, diversity, and arbovirus associations. PLoS One. 2013;8:e77017. 10.1371/journal.pone.0077017 24146951 PMC3795637

